# Squeezing an Egg into a Worm: C. elegans Embryonic Morphogenesis

**DOI:** 10.1100/tsw.2003.123

**Published:** 2003-12-18

**Authors:** A. J. Piekny, P. E. Mains

**Affiliations:** Genes and Development Research Group, Department of Biochemistry and Molecular Biology, University of Calgary, 3330 Hospital Dr. NW, Calgary, AB, Canada T2N 4N1,

**Keywords:** C. elegans, development, morphogenesis, actin, myosin, Rho-binding kinase

## Abstract

We review key morphogenetic events that occur during Caenorhabditis elegans (www.wormbase.org/) embryogenesis. Morphogenesis transforms tissues from one shape into another through cell migrations and shape changes, often utilizing highly conserved actin-based contractile systems. Three major morphogenetic events occur during C. elegans embryogenesis: (1) dorsal intercalation, during which two rows of dorsal epidermal cells intercalate to form a single row; (2) ventral enclosure, where the dorsally located sheet of epidermal cells stretches to the ventral midline, encasing the embryo within a single epithelial sheet; and (3) elongation, during which actin-mediated contractions within the epithelial sheet lengthens the embryo. Here, we describe the known molecular players involved in each of these processes.

## INTRODUCTION

Morphogenetic events transform an embryo from one shape into another. These changes originate at the cellular level and are based primarily on cell migrations and alterations in cell shape. The first major rearrangement is gastrulation (not covered in this review), where mesodermal cells migrate into the embryo resulting in three germ layers (see "gastrulation" www.bio.unc.edu/faculty/goldstein/lab/movies.html to view this process in *C. elegans*). Other types of morphogenetic movements important for embryogenesis include epiboly, where a sheet of epithelial cells extends across an embryo, and purse-string contractions (analogous to wound healing), where shortening at the apical edge of a group of cells surrounding an open space draws the cells together to close the space[1,2,3,4,5,6]. Critical to these processes are rearrangements of the actin cytoskeleton signaled by the Rho family of small Ras-like GTPases (Rho, Rac, and CDC42)[7,8,9,10]. These molecules regulate actin-based structures including filopodia, lamellipodia, and focal adhesions, which are critical for cell shape changes, movements, and the contractile process. In addition, adherent cell junctions maintain epidermal cells in an epithelial sheet to ensure that migrations and shape changes occur in a coordinated manner.

The nematode *C. elegans* is ideal for studying morphogenesis at the cellular level because the worm is transparent throughout its life cycle and all cell divisions can be viewed in living animals by Nomarski microscopy[11,12]. The small size (1.2 mm adults), short generation time (3.5 days at 20$^{o}$C), large brood size (>300 progeny), and hermaphroditic sexual system facilitate genetic analysis of developmental processes[13,14]. Genetic approaches have a crucial advantage over cellular and biochemical approaches as they can identify genes independent of the gene product's abundance. Genetics can also reveal the existence of redundant pathways that are difficult to detect by other methods. The complete genomic sequence of *C. elegans*[15] coupled with transformation rescue of mutant phenotypes allows rapid positional cloning of genes. In addition, RNA interference (RNAi)[16] efficiently disrupts the expression of genes lacking known mutations, allowing rapid analysis of candidate genes implicated by other systems.

In this review we will describe embryonic morphogenesis, which transforms the *C. elegans* embryo from a roughly spherical shape into a tube-like, vermiform larva. This transformation occurs in the absence of cell proliferation (reviewed in [5,6], and see “normal development” at www.bio.unc.edu/faculty/goldstein/lab/movies.html) and is driven by cell migration and shape changes within the surface layer of embryonic epidermal cells (also referred to as the “hypodermis” in nematodes)[17,18,19,20]. Approximately 300 min postfertilization, the epidermal cells are found in three pairs of rows straddling the posterior dorsal midline of the embryonic surface (Fig. 1). These cells will undergo three major morphogenetic events. Initially, the two rows of epidermal cells immediately adjacent to the dorsal midline intercalate with one another[17]. As dorsal intercalation completes, the ventral-most rows of epidermal cells undergo epiboly and migrate to the ventral midline to enclose the embryo within an epithelium (“ventral enclosure” www.bio.unc.edu/faculty/goldstein/lab/movies.html)[18]. On completion of ventral enclosure, an actin-mediated contraction of the lateral row of epidermal cells causes these cells, and the embryo, to contract, resulting in a fourfold longitudinal lengthening of the embryo[19]. The vermiform shape is then maintained by secretion of a cuticle by the epidermal cells[20]. We will now discuss each of these morphogenetic events in terms of morphology and the regulatory molecules involved in each event (all genes that will be discussed are summarized in Table 1).

## DORSAL INTERCALATION

Dorsal intercalation, the first major morphogenetic event occurs when the two rows of epidermal cells that flank the dorsal midline merge into a single row. These round cells become wedge shaped and extend basal processes (Figs. 1 and 2) that slide beneath the borders of neighboring cells toward the dorsal midline. Later, the cell bodies push their way into the new intercalated position to form a single row on the dorsal midline[17], and many of these cells fuse with one another.

Relatively few genes are known to be specifically involved in dorsal intercalation, possibly because the genes involved are essential for earlier morphogenetic events such as gastrulation (causing mutant embryos to die before displaying dorsal intercalation defects). In addition, redundant pathways may govern dorsal intercalation, precluding the identification of any single gene that is found in one or the other pathway.

*die-1* (dorsal intercalation and elongation defective) encodes a C2H2 zinc finger transcriptional regulator required for proper dorsal intercalation[21]. In *die-1* mutants, dorsal epidermal cells initiate intercalation properly, but the cell bodies fail to migrate. While ventral

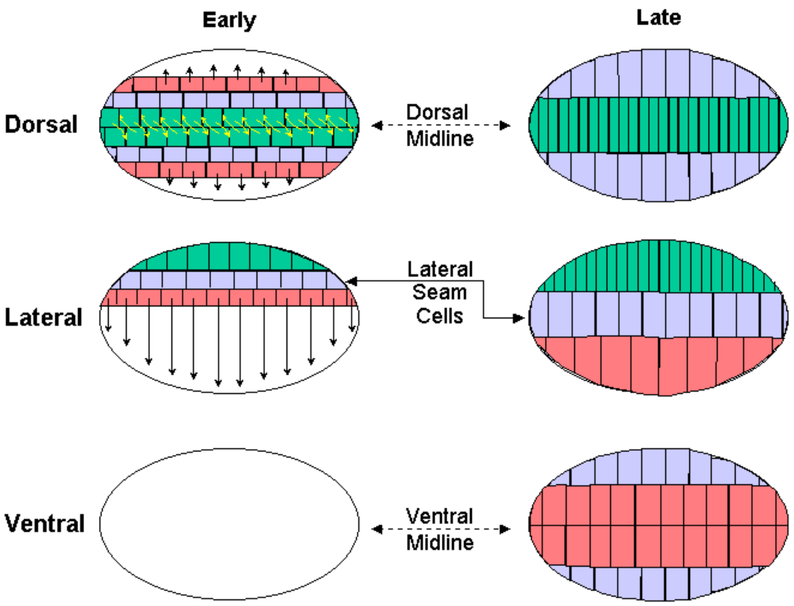

**FIGURE 1.** Schematic representation of the cell movements during dorsal intercalation and ventral enclosure. Initially, each side of the ventral midline contains three rows of epidermal cells. The dorsal-most rows (green) slide past one another as indicated by the arrows during dorsal intercalation, forming into a single row. Later, the ventral row (red) on either side of the dorsal midline migrates as indicated by the arrows to the ventral midline, enclosing the embryo.

enclosure is not defective, elongation fails. Pharynx, intestine, and muscle, which undergo embryonic morphogenetic changes similar to those in the epidermis, are also defective in *die-1*, suggesting that these morphogenetic events may utilize similar molecular mechanisms.

Intercalation movements fail in the *C. elegans* mutants of *gex-2* (gut on the exterior) and *gex-3*: epidermal cells remain bunched on the dorsal surface of the embryo and ventral enclosure does not occur (see below)[22]. Dorsal intercalation similarly fails when wild-type embryos are treated with cytochalasin D (an inhibitor of actin polymerization), indicating that the actin cytoskeleton is essential for this process[19]. Indeed, the Arp2/3 (actin related protein) complex, a complex of seven polypeptides that nucleates actin polymerization in eukaryotes, is also required for intercalation movements and ventral enclosure in *C. elegans*[23]. In other eukaryotes, the protein WASP positively regulates actin polymerization via Arp2/3[24]. WASP contains protein motifs that bind various signaling molecules, such as Cdc42, a Rho-GTPase[24]. Perhaps related to this, the homologues of *gex-2* and *gex-3* interact with the Cdc42-related small GTPase RacA; RacA is a mediator of the actin-based lamellipodia required for cell migration in cultured fibroblast cells[10,22]. The protein products of the *C. elegans* genes, denoted GEX-2 and GEX-3, localize to cell boundaries and could mediate Rac signaling required for epidermal cell migration, although how they affect Rac is currently unknown[22].

## VENTRAL ENCLOSURE

The next major morphogenetic event, ventral enclosure, begins prior to completion of dorsal intercalation ("ventral enclosure" at www.bio.unc.edu/faculty/goldstein/lab/movies.html). On each side of the embryo, the ventral and lateral rows of epidermal cells stretch and migrate to the ventral midline to enclose the embryo in a single continuous epithelial sheet whose integrity is critical for elongation of the embryo at a later stage (Figs. 1 and 2)[18]. Initially, two pairs of anterior ventral ("leading edge") cells extend filopodia and migrate ventrally over underlying

**TABLE 1**
**Genes Required for Morphogenesis**

| Gene [Ref.] | Protein | Morphogenetic Event |
|---|---|---|
| *die-1* [21] | C2H2 zinc finger transcription factor | Dorsal intercalation |
| *gex-2* [22] | Rac GTPase interacting protein | |
| *gex-3* [22] | Rac GTPase interacting protein | |
| *arp2/3* [23] | Microfilament nucleation | |
| *vab-1* [25] | Ephrin receptor tyrosine kinase | Ventral enclosure |
| *efn(1-4)* [26,27] | Ephrin ligands | |
| *mab-20* [29] | Semaphorin-2a | |
| *ptp-3* [32] | LAR-like protein tyrosine phosphatase | |
| *arp2/3* [23] | Microfilament nucleation | |
| *gex-2* [22] | Rac GTPase interacting protein | |
| *gex-3* [22] | Rac GTPase interacting protein | |
| *ced-10* [33] | Rac GTPase | |
| *hmr-1* [35] | Cadherin | |
| *hmp-1* [35] | α-Catenin | |
| *hmp-2* [35] | β-Catenin | |
| *let-413* [45,49] | GTPase adaptor protein | Elongation |
| *dlg-1* [46,47,49] | MAGUK | |
| *ajm-1* [48] | Novel coiled coil protein | |
| *sma-1* [50] | $\beta_H$-Spectrin | |
| *spc-1* [51] | α-Spectrin | |
| *hmr-1* [35] | Cadherin | |
| *hmp-1* [35] | α-Catenin | |
| *hmp-2* [35] | β-Catenin | |
| *mlc-4* [56,62] | Nonmuscle myosin light chain | |
| *nmy-1, nmy-2* [57] | Nonmuscle myosin heavy chain | |
| *let-502* [57,58,59,60] | Rho-binding kinase | |
| *mel-11* [57,58,59,60] | Myosin phosphatase | |
| *fem-2* [60] | PP2c phosphatase | |
| *mig-2* [59,60] | Rho/Rac-like GTPase | |
| *ced-10* [unpubl. obs.] | Rac GTPase | |
| *unc-73* [59,60] | Rho/Rac exchange factor | |
| *age-1* [60] | PI3 kinase | |
| *daf-2* [60] | Insulin receptor | |
| *daf-16* [60] | Forkhead transcription factor | |
| *pat* [63,64,65] | Muscle or hemidesmosome components | |
| *sqt-3* [19] | Collagen | Postelongation |

neuroblasts. Contralateral leading edge cells meet at the ventral midline and form adherens junctions (AJs, apically located electron dense structures important for linking cells into a single sheet). The ventral surface of the embryo becomes fully enclosed as a purse-string contraction draws the ventral “pocket” cells, which lie posterior to the leading edge cells, to the ventral midline.

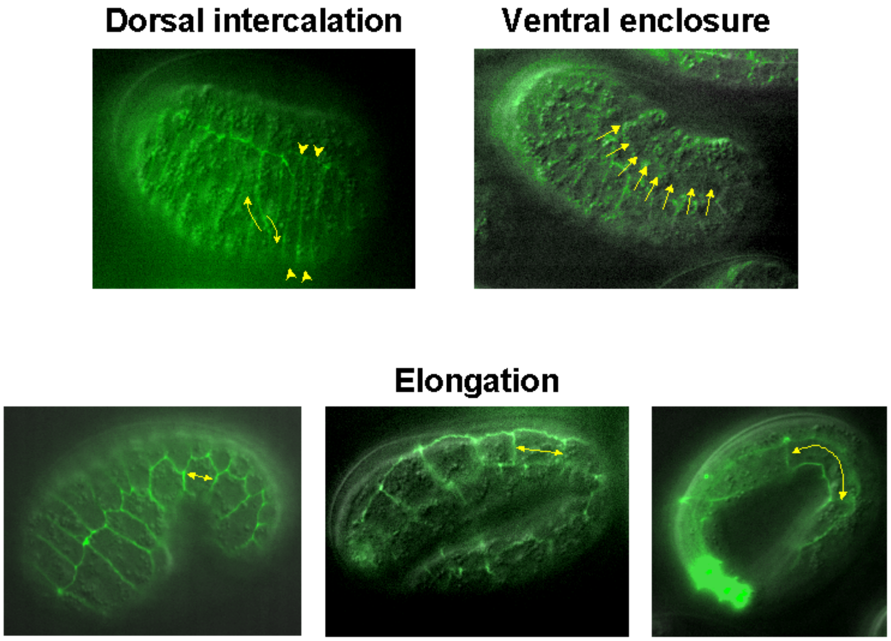

**FIGURE 2.** Nomarski images of living embryos expressing *AJM-1::GFP* (green fluorescent protein) at different morphogenetic stages. *AJM-1::GFP* (green) outlines the epidermal cells. A dorsal/lateral view of an embryo during the latter stages of dorsal intercalation is shown [top left]. A pair of wedge-shaped intercalating dorsal cells are moving past one another as indicated by the arrows. The adjacent pair of fully interdigitated cells are marked by the arrowheads. A lateral view of an embryo undergoing ventral enclosure is shown at the top right. The leading edge ventral epidermal cells are migrating toward the ventral midline as indicated by the arrows. Lateral views of embryos at various stages of elongation are shown in the bottom row. An arrow in a representative lateral epidermal "seam" cell at each stage highlights how these cells are lengthening along the anterior/posterior axis as elongation progresses (the bright anterior GFP signal is the pharynx).

The *vab-1* (variable abnormal) and *efn* (ephrin) genes define the migratory path of the epidermal cells. In anterior regions of *vab* or *efn* mutant embryos, the leading edge cells send out abnormal filopodia and migrate slowly or not at all, or migration to the ventral midline occurs but AJ formation fails[25,26,27]. These mutants also have incompletely penetrant defects in the closure of the gastrulation cleft and head and tail morphogenesis. *vab-1* and *efn 1* through *4* encode homologues of Eph receptor tyrosine kinase and ephrin, respectively, which in other organisms are involved in axon guidance, cell migration, and adhesion[25,26,27,28]. Recent experiments have linked Eph signaling to changes in the cytoskeleton through Rho GTPases[28]. However, *vab-1* and *efn-1* are both expressed in neuroblasts and function nonautonomously to affect epidermal morphogenesis.

Genetic experiments suggest that *efn-4* could function in a pathway with *mab-20* (male abnormal), which encodes Semaphorin-2a, although how this would occur still remains to be investigated[27,29]. *mab-20* is required to prevent ectopic filopodia formation during ventral enclosure[29]. Normally, only anterior leading edge cells extend filopodia during ventral enclosure[8]. However, in *mab-20* mutants the posterior ventral pocket cells also send out actin-rich processes. These cells develop inappropriate contacts that fail to form AJs with their contralateral neighbors causing embryos to rupture at the ventral midline later during elongation[29]. In higher eukaryotes, semaphorins sometimes function as repulsive cues, promoting axon growth cone collapse and repulsion. Recent work has linked the semaphorin Plexin receptors to Rac signaling[30]. Rac and other Rho family GTPases can promote or inhibit filopodia formation[8,9,10,31]. Semaphorin-2a might signal Rac to inhibit filopodia formation in the posterior ventral pocket cells, while still allowing filopodia to form in the more anterior leading edge cells. In addition, *ptp-3,* which encodes a member of the LAR subfamily of receptor tyrosine phosphatase, displays variable abnormal phenotypes similar to *vab-1* and the *efn*s[32]. *ptp-3* likely is involved in another, partially redundant pathway with *vab-1* and *efn-4/mab-20*, and together they ensure the careful coordination of epidermal cell migration over the underlying neuroblasts during ventral enclosure[27].

Consistent with a general role for the Rac system in cell movement, the Arp2/3 complex, *gex-2*, *gex-3*, and *ced-10* (cell death abnormal, a Rac homolog) display ventral enclosure defects. Epidermal cells either fail to migrate to the ventral midline or fail to form stable contacts causing embryos to rupture and organs, including the gut, are externalized[22,23,33]. Products of all three genes localize to epidermal cell boundaries during wild-type morphogenesis[22,34].

Linkage of the actin cytoskeleton to the membrane is required for formation of stable contacts between contralateral neighbors once they meet at the ventral midline. Classic catenin/cadherins link the actin cytoskeleton to the membrane and are important for ventral enclosure. Cadherins contain extracellular domain(s) for $Ca^{2+}$-dependent homotypic interactions and intracellular domain(s) that interact with β-catenin. β-Catenin in turn links cadherin to α-catenin, which directly interacts with actin[35]. In *C. elegans*, *hmp-1*, *hmp-2*, and *hmr-1* encode α-catenin, β-catenin, and cadherin, respectively[35]. *hmp* (humpback) mutants display abnormal bulging on their dorsal surface while *hmr* (hammerhead) fails to fully enclose and bulge at the anterior surface. Although loss of zygotic *hmp-1* or *hmp-2* results in elongation defects (see below), simultaneous loss of both maternal and zygotic *hmp-1* or *hmp-2* causes ventral enclosure defects similar to those observed in zygotic *hmr-1* mutants. For these latter mutants, ventral epidermal cells initiate migration correctly, but the anterior leading edge cells may fail to form initial fine contacts with their contralateral neighbors, and the cells never reach the ventral midline. Embryos subsequently rupture during elongation. The products of the three genes localize to AJs when the leading ventral epidermal cells meet at the ventral midline, suggesting that one of the roles of catenin/cadherin is the formation and/or stabilization of contralateral cell-cell contacts required for final enclosure of the embryo.

As described earlier, enclosure is completed when the posterior ventral pocket cells are drawn together by a purse-string contraction similar to those described in *Drosophila* dorsal closure and wound healing in higher eukaryotes. In *Drosophila*, leading edge epidermal cells elongate and extend filopodia over the underlying amnioserosa toward the dorsal midline and enclose the embryo after a purse-string contraction draws them together[4,10]. The apparent similarities between *Drosophila* dorsal closure and *C. elegans* ventral enclosure may indicate evolutionary conservation of morphogenetic mechanisms. However, while Rho family signaling likely mediates the rearrangement of the actin cytoskeleton in both species, Rho has unique roles in *Drosophila* that may not be conserved during *C. elegans* ventral closure. In addition to its effects on actin, Rho also regulates gene expression[8,9,10]. For example, in leading edge cells of the fly, Rho activates the Jun amino-terminal kinase (Jnk) signaling cascade to increase Dpp transcription, and Dpp (a TGF β-related molecule) in turn signals to neighboring cells, coordinating cell shape changes during dorsal closure[10,36,37,38,39,40]. The *C. elegans* homologues of Jnk and Dpp pathways apparently are not involved in ventral enclosure[41,42]. Likewise the genes employed for migratory cues in the worm (Eph and ephrin, Semaphorin-2a) play no known role in *Drosophila* dorsal closure[2,4]. Therefore, only downstream pathways directly regulating the actin cytoskeleton may be conserved between fly dorsal closure and worm ventral enclosure.

## ELONGATION

Following ventral enclosure, dramatic actin-mediated cell shape changes occur in the lateral epidermis, resulting in a fourfold lengthening of the embryo. Priess and Hirsh[19] found that circumferential filamentous bundles of actin (CFBs) form in the lateral epidermal (“seam”) cells immediately after ventral enclosure. CFB contraction causes these cells, and the embryo as a whole, to decrease in diameter with a concomitant lengthening of the longitudinal axis (Figs. 2 and 3A)[19]. Treatment of embryos with cytochalasin D disrupts the organization of CFBs and prevents elongation[19]. Many genes are required for elongation and reveal a complex process requiring the coordination of different events and different tissues.

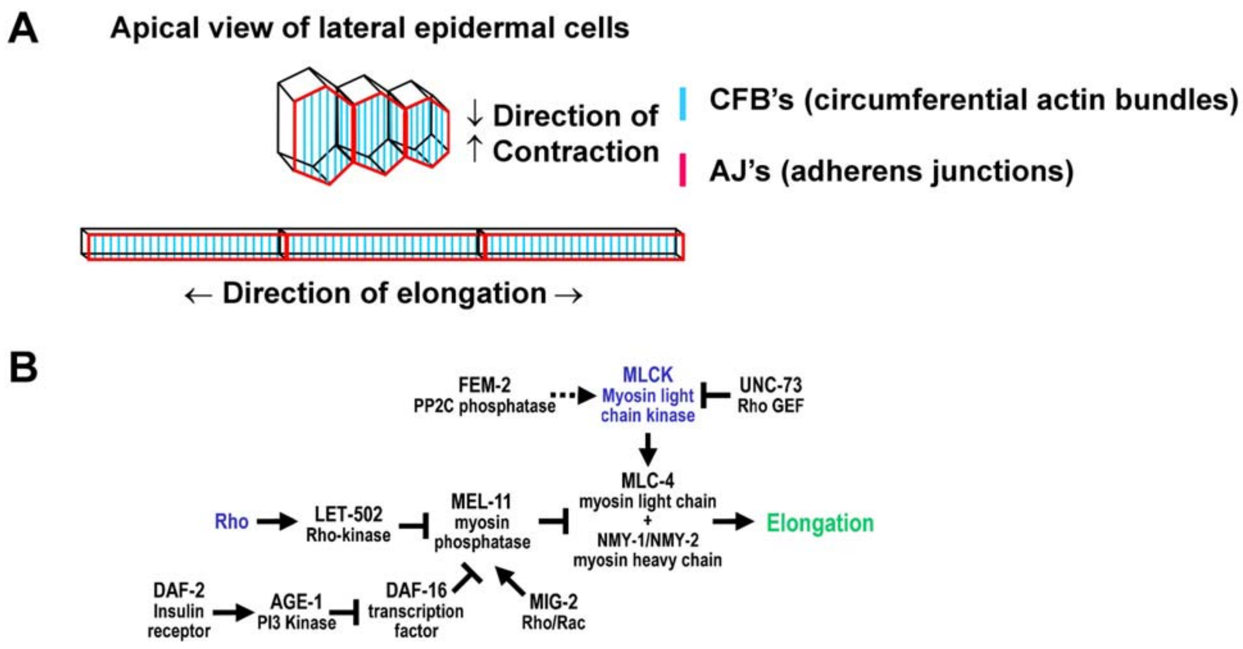

**FIGURE 3**. (A) A schematic of lateral epidermal "seam" cell shape changes during elongation. The neighboring seam cells are shown at the onset of elongation, and arrows indicate the direction of CFB (in blue) contraction. Seam cells change shape, becoming longer in their longitudinal axis with a concomitant transverse shortening, resulting in a shape change in the direction of the arrows. Neighboring seam cells are linked by AJs (red). (B) The pathway of genes mediating elongation. These interactions were determined by genetic and/or molecular experiments. Positive and negative interactions are indicated by pointed and barred arrows, respectively. Genes not yet shown to interact in the *C. elegans* pathway are colored in blue and are placed based on analogies with other systems. Dashed arrows indicate that the interactions may not be direct.

AJs play a critical role in transmitting the forces of contraction. These structures anchor CFBs to the seam cells' apical surfaces, and other AJs link all of the epidermal cells in a continuous epithelial sheet, allowing the uniform transmission of the contractile force across the embryonic surface[19,43,44]. Microtubules likely act as struts over which the contractile forces stretch the epidermis since disruption of microtubules with nocodazole prevents elongation[19].

*let-413* (lethal, a homolog of *Drosophila Scribble*, an adaptor protein that may interact with small GTPases), *dlg-1* (disks large, a homolog of *Drosophila disks large*, a MAGUK membrane-associated guanylate kinase-family scaffolding protein), and *ajm-1* (apical junction molecule) all are required for AJ formation, and mutants disrupt elongation[45,46,47,48,49]. LET-413 may define the basolateral border by directing or refining the apical localization of AJ proteins such as DLG-1 and AJM-1[45,46,47,48]. In *let-413* mutants, DLG-1 and AJM-1 mislocalize to basolateral positions resulting in polarity defects, improper AJ formation, and disorganization of the actin cytoskeleton[49]. Cells of these mutants cannot coordinately change shape, and embryos display elongation defects, often rupturing ventrally. Although *ajm-1* and *dlg-1* mutant embryos have wild-type actin organization, they fail to elongate, likely because defective AJs result in improper cell association during CFB contraction[45,46,47,48].

*sma-1* (small, $\beta_H$-spectrin) and *spc-1* ($\alpha$-spectrin) encode spectrin proteins that anchor CFBs to AJs[50,51]. *sma-1* null mutants are viable, but their slowed elongation rate results in short animals[50]. *spc-1* mutants are lethal and reach only the point of midelongation (twofold stage, i.e., when the uncurled embryo would be twice the length of the eggshell)[51]. Zygotic loss of *hmp-1*, *hmp-2*, and *hmr-1* catenin/cadherins result in elongation arrest at stages similar to the spectrins, with the CFBs pulling away from cell membranes[35]. HMP-1, HMP-2, and HMR-1 localize to AJs where they anchor the CFBs (Fig. 3A). Since *Drosophila* $\beta_H$-spectrin is found at AJs, worm spectrins may act similarly and could function in concert with catenins/cadherins to link the actin cytoskeleton to the membrane[50,51,52].

Once the intact epithelium is in place and CFBs are anchored, contraction drives the cell shape changes required for elongation. The actin-based motor complex responsible for CFB contraction is likely thick filaments composed of nonmuscle myosin, which is a hexamer of two heavy chains, two regulatory light chains (rMLC), and two essential light chains[53]. By analogy with smooth muscle contraction and focal adhesion formation in vertebrates, myosin contraction is likely induced by rMLC phosphorylation[8,9,10,53,54]. On activation, myosin light chain kinase (MLCK) or integrin-linked kinase (ILK) phosphorylates rMLC at Ser-19 (Ser-18 in *C. elegans*) and triggers contraction (Fig. 3B). Myosin phosphatase counteracts MLCK activity by dephosphorylating rMLC, resulting in relaxation. A Rho signaling pathway regulates contraction through its effector, Rho-binding kinase (ROK). At the appropriate time Rho enhances ROK activity, which either phosphorylates myosin phosphatase to render this inhibitor of contraction inactive, and/or directly phosphorylates rMLC. This results in higher levels of phosphorylated rMLC and thus favors contraction[53,54,55].

Mutations in worm homologues of genes in this contractile system indicate that the same pathway operates during *C. elegans* elongation. *mlc-4* (rMLC) zygotic null mutants arrest at the twofold stage of elongation due to failed CFB contraction[56]. The two *C. elegans* nonmuscle myosin genes, *nmy-1* and *nmy-2,* act redundantly during elongation and are thus the likely myosin heavy chain partners for *mlc-4* during embryonic morphogenesis[57].

The *C. elegans* homologues of ROK (LET-502) and myosin phosphatase (MEL-11) are required for embryonic elongation[57,58,59,60]. *let-502* mutants fail to elongate, consistent with ROK/LET-502 inducing CFB contraction. In contrast, *mel-11* mutants hyperelongate, likely due to unregulated CFB contraction[58,59,60]. LET-502 is highly expressed within the cytoplasm of elongating epidermal cells and localizes near components of the contractile apparatus. However, MEL-11 is initially cytoplasmic, but becomes sequestered away from the contractile apparatus during elongation in a LET-502-dependent manner[57]. The ROK-dependent localization change of myosin phosphatase also occurs during smooth muscle contraction, likely to prevent inhibition of the contractile machinery[61].

*let-502* and *mel-11* null mutants suppress one another, indicating that when both functions are removed, embryos elongate almost normally[58,59,60]. If the pathway was linear, one would expect to see the downstream phenotype (*mel-11*) in the double mutant; instead the double mutants grow to adults, albeit sterile and lumpy/dumpy in appearance. This implies that another pathway functions to regulate elongation in the absence of both *let-502* and *mel-11;* defects are only seen when the two activities are out of balance[60]. We have identified the PP2c phosphatase sex determination gene *fem-2* (<u>fem</u>inization) as a player in this redundant pathway, although other sex determination genes are apparently not involved in elongation[60]. *fem-2* mutants display low penetrance elongation defects; however, triple mutants with *let-502* and *mel-11* display severe elongation defects, presumably because both the *let-502/mel-11* and the parallel *fem-2* pathways are inactivated (Fig. 3B).

Several genes that regulate the worm actin cytoskeleton in other processes genetically interact with *mel-11* during elongation, although their effects are relatively weak. These genes include *mig-2* (Rho/Rac-like), *ced-10* (Rac), and *unc-73* (Rho/Rac guanine exchange factor)[59,60; P. Mains, unpublished]. Members of the insulin signaling pathway also interact genetically with *mel-11*; these include *age-1* (PI3 kinase), *daf-2* (insulin receptor), *daf-16* (forkhead transcription factor)[59,60]. Double and triple mutant analysis in combination to analogies with other systems led to the development of the model in Fig. 3B.

LET-502 and MEL-11 appear to function as part of a contractile cassette that is utilized for other contractile events within *C. elegans* embryos. Maternal LET-502 and MEL-11 localize to cleavage furrows where they regulate the rate of furrow ingression during cytokinesis[62]. As during elongation, *mlc-4* encodes the myosin light chain that interacts with LET-502/MEL-11; cytokinesis fails when *mlc-4* activity is removed[56], and MLC-4 phosphorylation levels change in the predicted manner in *let-502* and *mel-11* mutants[62]. Another similarity between cytokinesis and elongation is that *let-502* and *mel-11* suppress one another in both instances, and the pathways are redundant for both elongation and cytokinesis[62]. However, there are differences between cytokinesis and elongation as the other genes in Fig. 3B play no apparent role in cytokinesis, and genes that genetically interact with *let-502* during cytokinesis do not affect elongation. Both *let-502* and *mel-11* are also employed during spermathecal contractions, but they are expressed in different tissues and do not suppress one another[59]. Therefore, the core *let-502*/*mel-11* contractile cassette is used repeatedly throughout development, but accessory proteins differ between processes.

Although its mechanistic link to elongation is unclear, activation of muscle twitching is required for elongation. Longitudinal bands of muscles underlie the epidermis and form contacts, termed hemidesmosomes, with the epidermal basal lamina. Near the beginning of elongation, muscle myofilaments organize and begin twitching. Mutations in genes with a "Pat" phenotype (paralyzed arrest at two-fold) do not twitch and either disrupt muscle formation, function, or hemidesmosomal anchoring to the epidermal cells. For example, *pat-4*, which encodes ILK, displays a muscle phenotype due to failed hemidesmosome formation[63]. It is not clear why muscle twitching is important for elongation; perhaps nonactive muscle forms a passive mass that impedes elongation or active muscle helps to stretch the expanding epidermis or plays an inductive role with the overlying epidermis[64]. An intriguing possibility is that the muscle attachment structures may induce proper alignment of the CFBs in the epidermal cells[65]. Regardless, both the epidermis and the muscle are essential for elongation; neither alone is sufficient.

## POST ELONGATION — MAINTAINING SHAPE

The cuticle is secreted from the apical surface of the epidermal cells, and this maintains the vermiform shape. Treatment of the embryo with cytochalasin D during elongation causes the embryo to retract in length; however, addition of the drug after elongation is completed has no effect, presumably because the rigid cuticle has locked in the shape[20]. Cuticle-defective mutations in the collagen gene *sqt-3* (squat) elongate normally but are unable to maintain their shape[19].

## SUMMARY

*C. elegans* embryos undergo several morphogenetic events that transform the embryo from a ball of cells into the familiar worm shape. These events involve cell movements to encase the embryo, force-generating cell shape changes and formation of an intact epithelial sheet to transmit those forces across the embryonic surface. We are just beginning to understand the regulation of these morphogenetic events, but new genes involved in *C. elegans* embryonic morphogenesis are continuously being uncovered. Many of the genes regulating the actin cytoskeleton are highly conserved among eukaryotes, including members of the Rho gene family, their regulators, and effectors, and these play critical roles in *C. elegans* morphogenesis. Since mechanisms analogous to embryonic morphogenesis are utilized elsewhere during *C. elegans* development and are also employed in other organisms for process like filopodia formation, cell migration, axon outgrowth, and smooth muscle contraction, study of one system will undoubtedly shed light on many others.
